# Single-Step Fabrication of Computationally Designed Microneedles by Continuous Liquid Interface Production

**DOI:** 10.1371/journal.pone.0162518

**Published:** 2016-09-08

**Authors:** Ashley R. Johnson, Cassie L. Caudill, John R. Tumbleston, Cameron J. Bloomquist, Katherine A. Moga, Alexander Ermoshkin, David Shirvanyants, Sue J. Mecham, J. Christopher Luft, Joseph M. DeSimone

**Affiliations:** 1 Joint Department of Biomedical Engineering, University of North Carolina at Chapel Hill and North Carolina State University, Chapel Hill, North Carolina, United States of America; 2 Department of Molecular Pharmaceutics, University of North Carolina at Chapel Hill, Chapel Hill, North Carolina, 27510, United States of America; 3 Carbon, Redwood City, California, United States of America; 4 Department of Chemistry, University of North Carolina at Chapel Hill, Chapel Hill, North Carolina, United States of America; 5 Lineberger Comprehensive Cancer Center Institute for Nanomedicine, University of North Carolina at Chapel Hill, Chapel Hill, North Carolina, United States of America; 6 Department of Chemical and Biomolecular Engineering, University of North Carolina at Chapel Hill, Chapel Hill, North Carolina, United States of America; Kyoto Daigaku, JAPAN

## Abstract

Microneedles, arrays of micron-sized needles that painlessly puncture the skin, enable transdermal delivery of medications that are difficult to deliver using more traditional routes. Many important design parameters, such as microneedle size, shape, spacing, and composition, are known to influence efficacy, but are notoriously difficult to alter due to the complex nature of microfabrication techniques. Herein, we utilize a novel additive manufacturing (“3D printing”) technique called Continuous Liquid Interface Production (CLIP) to rapidly prototype sharp microneedles with tuneable geometries (size, shape, aspect ratio, spacing). This technology allows for mold-independent, one-step manufacturing of microneedle arrays of virtually any design in less than 10 minutes per patch. Square pyramidal CLIP microneedles composed of trimethylolpropane triacrylate, polyacrylic acid and photopolymerizable derivatives of polyethylene glycol and polycaprolactone were fabricated to demonstrate the range of materials that can be utilized within this platform for encapsulating and controlling the release of therapeutics. These CLIP microneedles effectively pierced murine skin *ex vivo* and released the fluorescent drug surrogate rhodamine.

## Introduction

Microneedle patches are arrays of sharp, sub-millimeter-sized needles that enhance transdermal drug delivery by physically perforating the tough outer layer of the skin to enable a therapeutic of interest to more easily pass into the body. [1,2] Unlike hypodermic needles, microneedles are small enough that they avoid nerve endings buried deep within the skin; therefore, they provide pain-free drug delivery with the potential for self-administration. [1–3] Microneedles enable transdermal drug delivery of a range of therapeutics that are difficult to deliver orally, such as proteins,[4] nucleic acids, [5] and large, hydrophobic molecules.[6]

Microneedles have been fabricated from a variety of different materials such as metal,[7,8] silicon,[9,10] and natural [11] and synthetic [12,13] polymers. Although numerous materials have been utilized, microneedles fabricated from biocompatible materials are considered the gold standard for patient safety because they avoid immunological risks associated with accidental microneedle fragmentation within the skin.[11–13] Typically, an active pharmaceutical agent (API) is uniformly distributed throughout a natural or synthetic polymeric matrix which dissolves, swells, or degrades to release the API into the body. With appropriate matrix selection, many pharmacokinetic profiles are achievable, providing improved control of therapeutic concentrations to maximize efficacy while minimizing side effects. [11–13]

**Fig 1:**
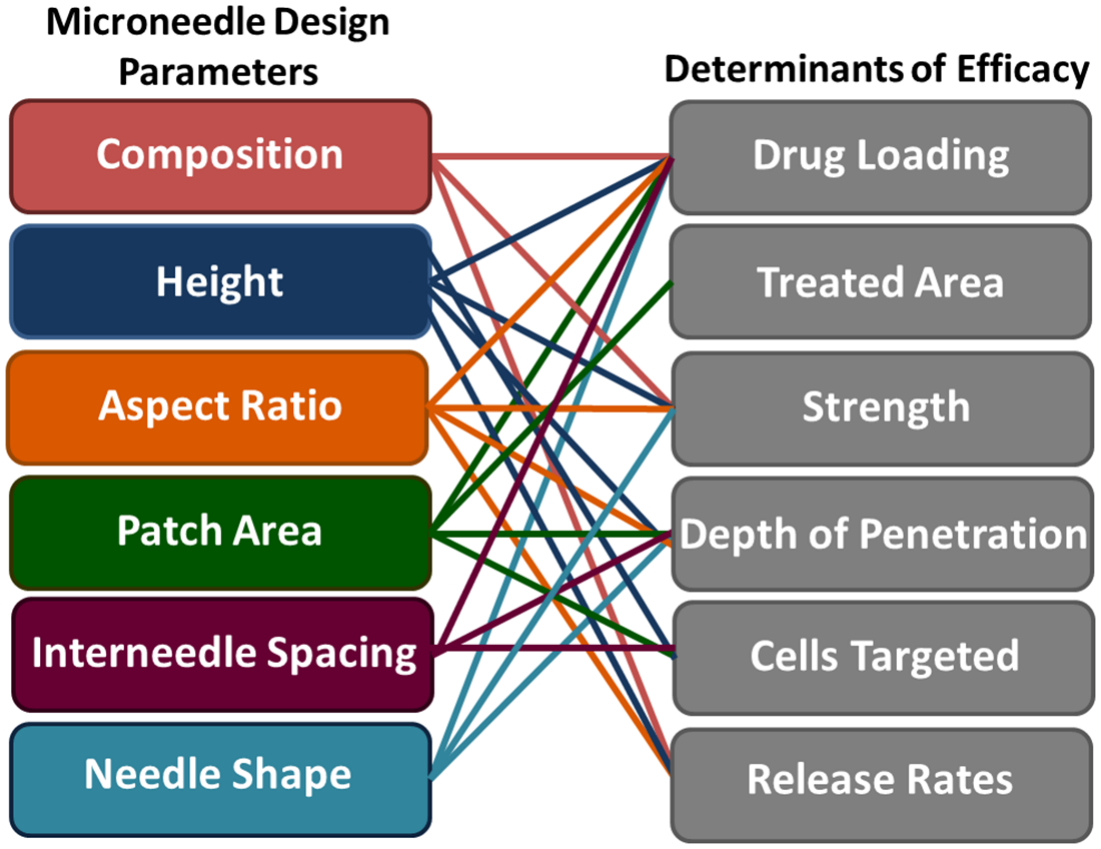
Relationship between microneedle design parameters and therapeutic efficacy. The left column is a list of microneedle design parameters, whereas the right column is a list of factors affecting the efficacy of microneedles used for transdermal drug delivery. Connections between input design parameters and their effect on efficacy are marked with solid lines.

Recently, a need for improved control over microneedle design parameters, including composition,[11,14–15] height,[16–19] sharpness,[20–21] aspect ratio,[22] inter-needle spacing, [23–25] and microneedle shape,[11,26] has been demonstrated. Such design parameters are known to influence microneedle efficacy (Fig 1). [14,19] For example, much work has been done to identify appropriate materials for microneedle fabrication. While metals are typically strong enough, biocompatible polymers must be carefully selected to have sufficient mechanical strength.[27] Because an efficacious microneedle must insert into the skin without breaking, both the force required for insertion and the failure strength of the material are important.[27] Microneedle shape, aspect ratio[22] and composition[11,14–15] dictate strength, whereas microneedle sharpness[20] and the number of needles in an array[24] influence insertion force.

Further, microneedle design parameters influence the total amount of a therapeutic that can be effectively delivered to the body. Because of their small size, the encapsulation and delivery of therapeutically relevant quantities of medication is challenging for anything but the most potent therapeutics.[28] For this reason (and others), clinical trials using microneedles have focused on the delivery of vaccines and hormones, which only require microgram levels to produce an efficacious response. Increasing microneedle height improves maximum cargo loading (due to increased microneedle volume), but microneedles that are too tall can be painful to patients.[16] Increasing the number of microneedles in an array also improves cargo loading, but the force required to insert the array also increases with the number of needles.[24–25] The design of efficacious microneedle patches is characterized by such trade-offs, complicating microneedle design. Developing a fundamental understanding of effective operating windows for each design parameter would aid the advancement of microneedle technology.

Ideally, new microneedle designs could be rapidly prototyped to systematically invesitgate each design parameter with the goal of optimizing efficacy. However, due to the complex nature of current microneedle fabrication techniques (such as silicon etching,[28,29] tilted UV (ultraviolet) photolithography,[11] and laser ablation[30] combined with micromolding), lead times for new designs are on the order of months. Further, many of these techniques have technical limitations that prevent certain types of microneedles (such as tall, sharp, and/or high aspect ratio structures) from being produced. For this reason, microneedle height, aspect ratio, and spacing are typically dictated by feasibility of fabrication rather than ideal design. A number of new microneedle fabrication techniques, such as drawing lithography,[31] two photon polymerization[32] and electrodrawing,[33] have been developed to address the limitations of traditional approaches, but are not widely adopted.

Additive manufacturing (“3D printing”) may provide a “touch-button” approach to computationally designing and rapidly prototyping microneedle patches. Additive manufacturing traditionally produces objects in a layer-by-layer fashion, wherein each layer is stacked on top of the previous layer to generate a three dimensional object (S1 Fig). In bottom-up stereolithography (a type of additive manufacturing), layers are generated by illuminating a vat of photoreactive resin through a UV transparent window underneath the resin. Layers then cure to the window through photopolymerization and must be mechanically separated and realigned before exposing the next layer (S2 Fig). Traditional stereolithography is not amenable to microneedle production because of poor resolution (each layer is typically ~100μm thick) [34] and because the large mechanical force required to separate objects from the window has a tendency to damage delicate parts.[35]

**Fig 2:**
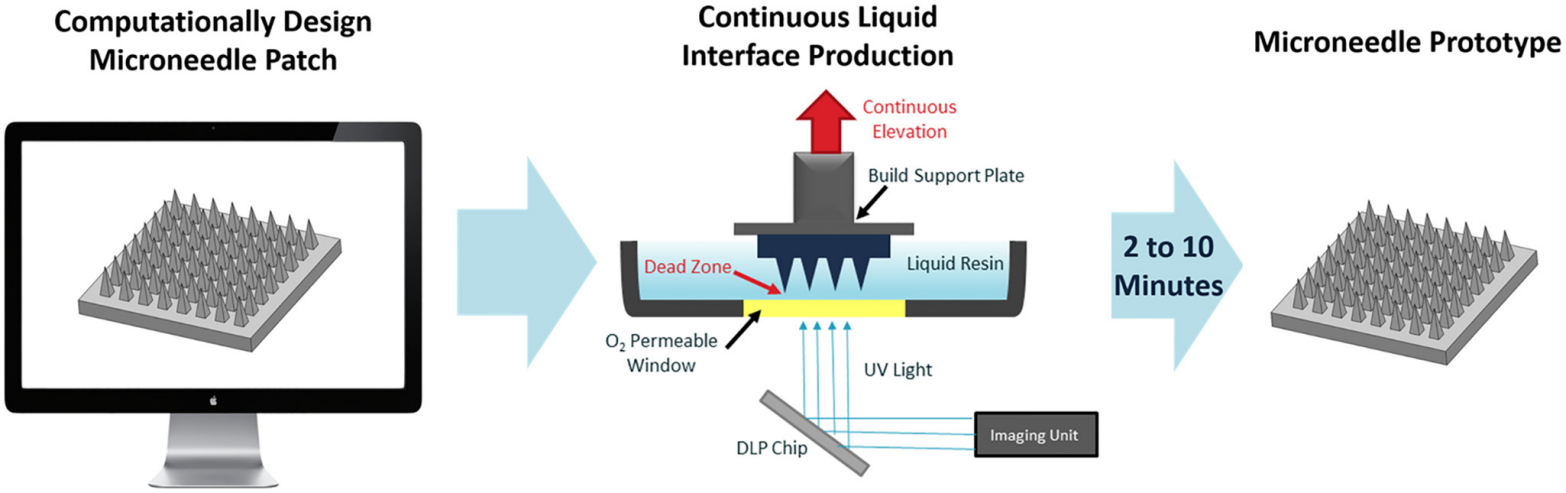
Continuous Liquid Interface Production (CLIP) Process. A microneedle patch is computationally designed using a CAD file. The microneedle is then fabricated using CLIP to produce a microneedle prototype within two to ten minutes. CLIP generates the microneedle patch through photopolymerization of a liquid, photoreactive resin using light reflecting off of a DLP chip. Continuous (rather than layer-by-layer) fabrication of the patch is enabled by a “dead-zone” created through oxygen mediated inhibition of the photopolymerization reaction at the window surface. A microneedle patch of virtually any design is created in two to ten minutes.

We have developed a new continuous, rather than layer-by-layer, approach to additive manufacturing called Continuous Liquid Interface Production (CLIP) [36] which is applied, here, to rapid prototyping of microneedle arrays. CLIP differs from stereolithography because it utilizes an oxygen permeable window, which inhibits photopolymerization at the window surface, to prevent the part from adhering to the window. Resin can then freely flow into this liquid “dead zone” at the window surface, enabling continuous rather than layer-by-layer production of the part to enable 1) faster production times by eliminating rate-limiting separation and realignment steps (Fig 2 and S2 and S3 Figs) generation of high resolution structures (such as microneedles) that would typically be damaged during traditional mechanical separation steps. [35]

Herein, we have utilized CLIP to rapidly prototype microneedles of a wide variety of sizes, shapes, and compositions with modular control over each design factor. We report rapid additive manufacturing of microneedle arrays from trimethylolpropane triacrylate and the biocompatible materials polyethylene glycol dimethacrylate, polycaprolactone trimethacrylate, and polyacrylic acid. The ability to adjust microneedle size and shape by altering a computer aided design (CAD) file is demonstrated. CLIP microneedles are shown to possess the chemical and mechanical properties necessary to penetrate murine skin and release the fluorescent drug surrogate rhodamine. We anticipate that the rapid and tunable nature of the CLIP technique will enable the high throughput, systematic investigation of parameters associated with microneedle design and accelerate translation of microneedle technology into a clinical setting.

## Materials and Methods

### Synthesis of poly-ε-caprolactone trimethacrylate (PCL-tMa)

Poly-ε-caprolactone (PCL) triol (Sigma Aldrich) with an average molecular weight of 900g/mol (55.14 g, 61.3 mmol) was dried in a vacuum oven. The reaction flask was equipped with an addition funnel, sealed with rubber septa, and placed under magnetic stirring and N_2_ flow. Distilled dichloromethane (DCM, 200mL, Fisher Scientific) and triethyamine (TEA, 275.9 mmol, Fisher Scientific) was added to a flask placed under magnetic stirring and N_2_ flow on an ice bath. Methacryloyl chloride (Sigma Aldrich) was added dropwise from the addition funnel over one hour and the reaction proceed overnight. The formed TEA•HCl salt was filtered off and the filtrate was washed with sodium bicarbonate, dried over magnesium sulfate, and the DCM was removed by rotary evaporation.

### Determination of Resin Cure Dosages

Methods for the determination of cure dosage were adapted from Tumbleston et. al.[32] Briefly, 500μL of resin was placed on a cover slip on top of the printer window. Resin was exposed to a specified dosage of light (λ = 365nm LED) in a circle pattern and residual monomer was removed using an acetone wash. The height of polymerized circles was then measured using a Mitutoyo Electronic Indicator (McMaster Carr). Resins utilized in this study were acrylic acid (Acros Organics, 99.5% purity), a poly-ε-caprolactone trimethacrylate (PCL-tMa) synthesized in house, and poly (ethylene glycol) dimethacrylate (M_n_ 550, Sigma Aldrich) mixed with 2.5 wt% Diphenyl(2,4,6-trimethyl-benzoyl-)phosphine oxide(TPO,Sigma Aldrich) as a photoinitiator.

### Fabrication of Trimethylolpropane Triacrylate (TMPTA) Microneedles

To produce microneedles of different heights, CAD files of square pyramidal microneedles measuring 1000, 700, and 400 μm tall with an aspect ratio of 3 (aspect ratio = height/width) were generated using Solidworks 2014. All microneedles were spaced at one base width apart on a base measuring 6x6x1mm. CAD files were then sliced at 1μm slice thickness using the open source software Slic3r. Microneedles were then produced using a CLIP additive manufacturing system (Carbon, Redwood City, CA) in a mixture of TMPTA (Sigma Aldrich) and 2.5wt% diphenyl(2,4,6-trimethylbenzoyl)phosphine oxide (Sigma Aldrich) with 5.4mW/cm^2^ of UV light (λ = 370nm LED) as measured by the Dymax AccuCal^™^50 (Dymax Corporation) at 100mm/hr. Z scale factors of 1.175, 1.175, and 1.6 were added during scaling to counteract z-axis truncation visualized without scaling. All microneedles were visualized using an environmental scanning electron microscope (FEI Quanta 200) in low vacuum mode.

To demonstrate ability to alter microneedle size and shape, CAD files were created and sliced as previously described. The CAD file used to generate microneedles of varying aspect ratios contained microneedles measuring 1000μm in height and 500μm, 333μm, and 250μm in width for aspect ratios of 2, 3, and 4, respectively. These microneedles were spaced at 333 μm apart. CAD files for microneedles of varying spacing measured 1000μm in height and 500μm in width with spacing at 250 μm or 500μm apart.

Arrowhead microneedles measuring 1000μm tall and 500μm wide were fabricated from TMPTA + 2.5wt% TPO (and 0.1wt% 2-(3’-tert-butyl-2’-hydroxy-5’-methylphenyl)-5-chlorobenzotriazole (Sigma Aldrich) at 41mm/hr with 5.4mW/cm^2^ of UV light. Tiered microneedles, measuring 1000μm, 800μm, and 600μm tall and 400μm wide, and turret microneedles measuring 1000μm tall and 500μm wide were fabricated at 25mm/hr with 1.35mW/cm^2^ of UV light.

### Biocompatible Microneedles

Poly-acrylic acid microneedles, polycaprolactone microneedles, and poly (ethylene glycol) microneedles were all printed at 25mm/hr, with 8.9, 1.5, and 1.2 mW/cm^2^ of light(λ = 370nm LED), respectively. All needles were washed briefly with acetone and dried using compressed air. Microneedles were imaged as previously described.

Microneedles containing multiple compositions were fabricated from polycaprolactone trimethacrylate mixed with 0.05wt% rhodamine B base (Sigma Aldrich) and acrylic acid mixed with 0.05wt% fluorescein (Sigma Aldrich). Both resins contained 2.5wt% TPO as a photoinitiator. Slices 1 through 1700 were fabricated using PCL prior to lifting the build elevator, removing residual resin, and continuing to fabricate the remainder of the microneedle (slices 1701 through 2000) using acrylic acid.

To test for acrylic acid microneedle dissolution, one patch with a polycaprolactone base and polyacrylic acid microneedles contining 0.1wt% rhodamine was submerged in 10mL PBS. The microneedle patch was imaged before and after dissolution using a Leica MZ16FA macroscope in brightfield mode.

### Skin Penetration Studies

Patches were tested on ex vivo nude murine skin with permission of the UNC Institutional Animal Care and Use Committee (IACUC). Nude mice were sacrificed through inhalation of isofluorane followed by cervical dislocation. Skin was then excised from the back and flank and all samples were stored at -20°C until testing occurred. Prior to testing, skin was thawed briefly at room temperature and pinned over corkboard. CLIP microneedles were post-cured under a mercury lamp for 5 minutes to improve mechanical strength prior to application. Microneedle patches were then applied to the skin with 10 seconds of thumb pressure before patch removal. A 50:50 mixture of Green tissue marking dye (Cancer Diagnostics) and isopropanol was then applied to the site for 3 minutes before being wiped away with water and isopropanol. Skin was imaged to visualize sites of microneedle insertion using brightfield macroscopy (Leica M420).

To further confirm skin penetration using histology, polyacrylic acid microneedles were applied to murine skin *ex vivo* with 10 seconds of thumb pressure before patch removal. Murine skin sections were then embedded in Tissue-Tec Optimum Temperature Cutting Medium (Sakura Finetek), bisected, and sectioned in 12 micron slices at -25°C (Leica Cryostat). Samples were H&E stained (Cryo-KIT, Cancer Diagnostics) and visualized using brightfield microscopy (Olympus BX61 Upright Brightfield Microscope).

To test for dye release, polyacrylic acid microneedle patches containing 0.1wt% rhodamine B were applied to murine skin *ex vivo* and left to dissolve in the skin for 30 minutes. Samples were then briefly fixed in FROZEN-FIX (Cancer Diagnostics) for 10 seconds and visualized using fluorescence microscopy (Olympus BX61 Upright Fluorescence microscope).

### Fabrication and Testing of Janus Microneedles

Tip-loaded microneedles were fabricated from polycaprolactone trimethacrylate mixed with 0.05wt% rhodamine B base (Sigma Aldrich) and acrylic acid mixed with 0.05wt% fluorescein (Sigma Aldrich). Both resins contained 2.5wt% TPO as a photoinitiator. Slices 1 through 1700 were fabricated using PCL prior to lifting the build elevator, removing residual resin, and continuing to fabricate the remainder of the microneedle (slices 1701 through 2000) using acrylic acid. Microneedles were post-cured under a mercury lamp for 10 min between compositions and after fabrication was complete.

To test for dye release from tip-loaded microneedles, tip-loaded microneedles were fabricated from blank polycaprolactone and a polyacrylic acid tip containing 0.1wt% rhodamine B base, as previously described. Microneedles were applied to murine skin *ex vivo* with gentle thumb pressure for a period of 10 seconds. Microneedles were then allowed to remain the the skin for a period of 30 minutes prior to imaging using a Leica MZ16FA macroscope in brightfield mode.

## Results

### Fabrication of TMPTA Microneedles of Different Sizes

To assess feasibility of applying CLIP to microneedle production, we investigated the fabrication of microneedles using a model resin composed of trimethylolpropane triacrylate (TMPTA mixed with 2.5wt% diphenyl (2,4,6- trimethylbenzoyl) phosphine oxide (TPO) as a photoinitiator. This model resin has been previously used in additive manufacturing because it is fast-reacting and has low viscosity;[37] therefore, the resin was used as a positive control for microneedle composition while establishing fabrication protocols.

**Fig 3:**
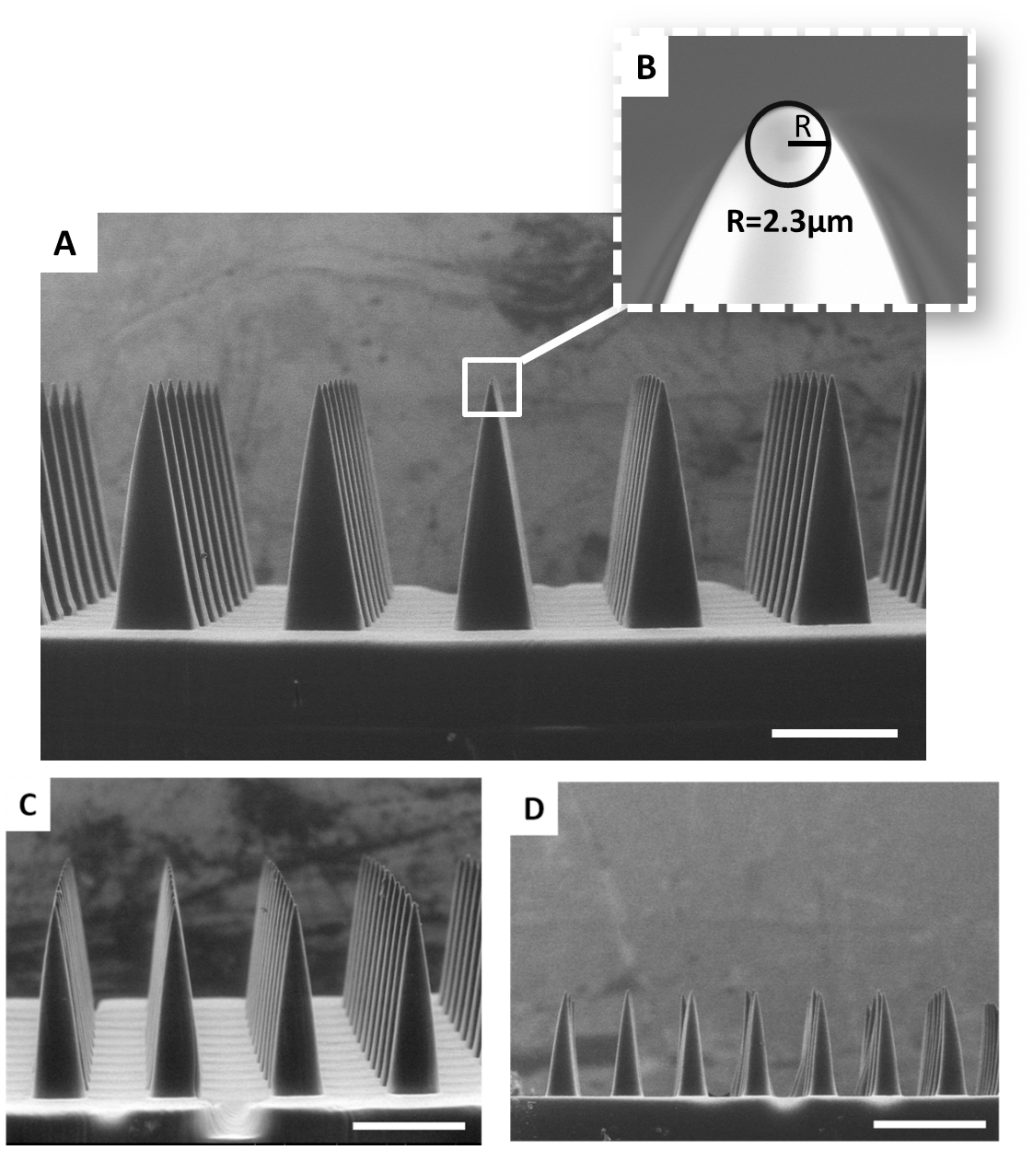
TMPTA Microneedles of Different Heights. TMPTA microneedles measuring approximately A) 1000μm C) 700μm and D) 400μm in height with AR = 3. B) Representative image of a microneedle tip with a tip radius of approximately 2.3μm. Scale bars measure 500μm (A, C-D) and 5μm (B), respectively. All patches were generated in less than 90 seconds.

A computer aided design (CAD) file of the desired microneedle array containing square pyramidal microneedles measuring 1000μm tall and 333μm wide, was generated and computationally sliced along the z direction. A digital light processing (DLP) chip and an ultraviolet light emitting diode (UV LED) were used to project these slices (frames) onto an oxygen permeable, UV-transparent window in rapid succession (Fig 2). This DLP chip is essentially an array of micromirrors which controls the shape of the UV light distribution passing through the window and onto the photoreactive resin. Microneedles were fabricated with varying amounts of UV light to determine how light intensity affects microneedle structure and dimensions (S4 and S5 Figs). Produced microneedles had the desired square pyramidal structure regardless of light intensity, but the size of the structure increased with increasing light intensity due to the increased amount of electromagnetic radiation available to initiate photopolymerization. Although all microneedles initially truncated in the z direction relative to the CAD file (regardless of light intensity), the addition of a z scale factor during fabrication allowed for the production of microneedles measuring within ±5% of the intended dimensions (Fig 3 and S1 Table).

Microneedles of different heights may be desirable for controlling depth of penetration in the skin and altering the volume available for cargo loading.[17,19] Therefore, CAD files of microneedles measuring 700μm and 400μm in height were also generated with aspect ratio held constant. Fig 3 shows that CLIP could be used to produce microneedles ranging from 400μm to 1000μm in height could with remarkable consistency across the array. Patches were fabricated in less than 90 seconds per patch with tip radii measuring less than 3.5 μm (Fig 3 and S1 Table).

### Fabrication of TMPTA Microneedles of Different Geometries

As previously mentioned, the geometry of a microneedle array is known to affect its cargo loading volume, failure force, [22] and ability to effectively insert into the skin,[24,25] among other factors. In order to determine whether CLIP can be utilized to rapidly adjust patch geometry, we fabricated CLIP microneedles with different aspect ratios and spacings.

**Fig 4:**
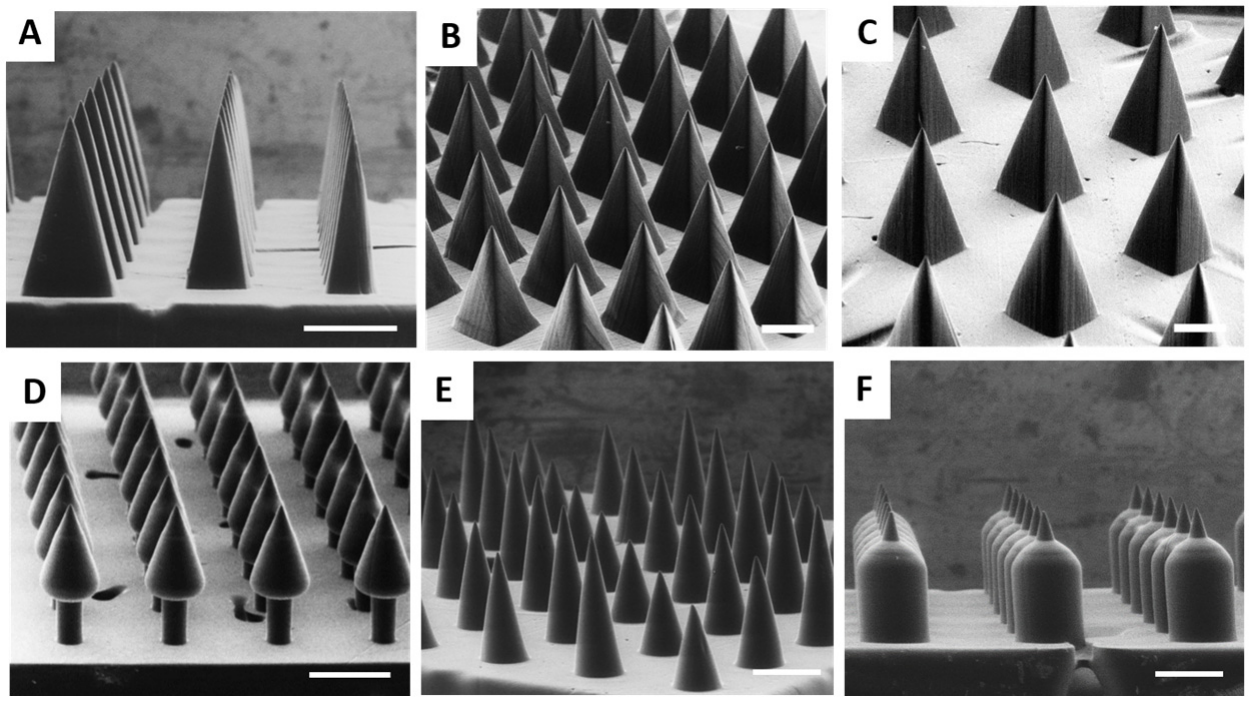
TMPTA Microneedles of Different Shapes. A) TMPTA microneedles of aspect ratio 2, 3, and 4 (left to right). 1000μm tall TMPTA microneedles with spacing of B) 0.5 base widths and C) 1.5 base widths. Complex microneedle geometries such as D) Arrowhead microneedles E) Tiered microneedles and F) Turret microneedles may improve mechanics of insertion into the skin. Scale bars measure 500μm.

The process of altering CLIP microneedle aspect ratio only requires generation of a new CAD file, as previously described. Fig 4A demonstrates the ability to simply adjust microneedle aspect ratio from 2 to 4 while maintaining desirable microneedle morphology. Similarly, CAD files of microneedles spaced at 0.5 and 1.5 base widths apart were readily generated and utilized to produce CLIP microneedle patches with adjustable inter-needle spacing (Fig 4B and 4C). Microneedle height was held constant at approximately 1000μm.

Although conical and square pyramidal microneedles have been the mainstay of microneedle technology, more complex geometries may afford improved penetration into the skin. For example, arrowhead microneedles may improve the consistency of needle penetration by resisting the elastic nature of the skin to remain embedded at their maximum penetration depth.[37] Successful microneedle penetration into the skin is also known to be inhibited by the “bed-of-nails” effect, wherein the total insertion force is divided evenly amongst every microneedle in an array, increasing the total force required for insertion.[24,25] The design of “tiered” microneedles, which contain microneedles of different heights on a single array, may reduce required insertion forces by concentrating the force on fewer needles at a given moment in time. Lastly, traditional square pyramidal microneedles of different aspect ratios are thought to present a trade-off between ease of insertion and microneedle strength wherein wider needles provide mechanical stability, but thinner needles more easily insert into the skin.[11–12] “Turret” microneedles containing sharp tips with a wide base may easily puncture the skin, but also afford improved mechanical strength.

CLIP was utilized to fabricate arrowhead microneedles, “tiered” microneedles, and “turret” microneedles, shown in Fig 4D–4F, respectively. This work demonstrates proof of concept that CLIP can be utilized to rapidly generate an almost infinite library of computationally defined microneedle geometries, which can be used to systematically investigate how geometry influences efficacy.

### Fabrication of Biocompatible Microneedles

After establishing techniques for microneedle fabrication using a model resin, we sought to fabricate microneedles from biocompatible materials designed for the incorporation and release of therapeutic cargos. The selection of an optimal microneedle matrix for a given application depends on the solubility of the cargo and the desired pharmacokinetic release profile. Therefore, we investigated fabrication of CLIP microneedles from materials with a range of solubility and release characteristics. Photopolymerizable derivatives of materials prevalent in FDA approved medical devices were selected to maximize biocompatibility.

Monomers selected for CLIP microneedle fabrication were poly(ethylene glycol) dimethacrylate (M_n_ = 550), polycaprolactone trimethacrylate (M_n_ = 1100) (PCL-tMa), and acrylic acid. Poly(ethylene glycol) (PEG) is a water-miscible polymer that has been utilized extensively for the delivery of protein based therapeutics;[38] the use of polyethylene glycol for microneedle fabrication has also been previously reported.[39] Photopolymerization of methacrylate functionalized PEG produces a crosslinked hydrogel that swells in aqueous environments. Drug release from the PEG hydrogel is likely to occur via diffusion out of the swellable matrix, with release rates dependent on various factors including the crosslink density of the hydrogel and the size, isoelectric point, and hydrophilicity of the cargo.[40] Poly(caprolactone) (PCL), which was modified for photopolymerization via addition of methacrylate endgroups, is a lipophilic material ideal for incorporating hydrophobic molecules (such as chemotherapeutics), which typically exhibit poor oral bioavailability.[36] PCL enables sustained release of lipophilic cargos through hydrolytic degradation of ester linkages in the polymer backbone. Acrylic acid (AA), which polymerizes to form linear polyacrylic acid (PAA), was selected as a water soluble matrix that is expected to rapidly dissolve upon insertion into the skin. All monomers were mixed with 2.5wt% TPO as a photoinitiator.

Appropriate selection of material-dependent build parameters, particularly light intensity and build speed, is essential to obtaining proper device morphology using the CLIP process. In order to rapidly establish appropriate build parameters for the biocompatible microneedles, we used a “working curve”, adapted from Tumbleston et. al.[36] This “working curve” normalizes for differences in reaction kinetics between resins by relating the cure height of the resin during a single frame to the intensity and duration of light exposure (for more information see SI, S7 Fig). In this way, build parameters utilized to fabricate TMPTA microneedles could be mathematically converted to build parameters appropriate for microneedle fabrication with biocompatible materials. CLIP could then be utilized as a “plug-and-play” approach to fabricate microneedles from a range of different materials.

**Fig 5:**
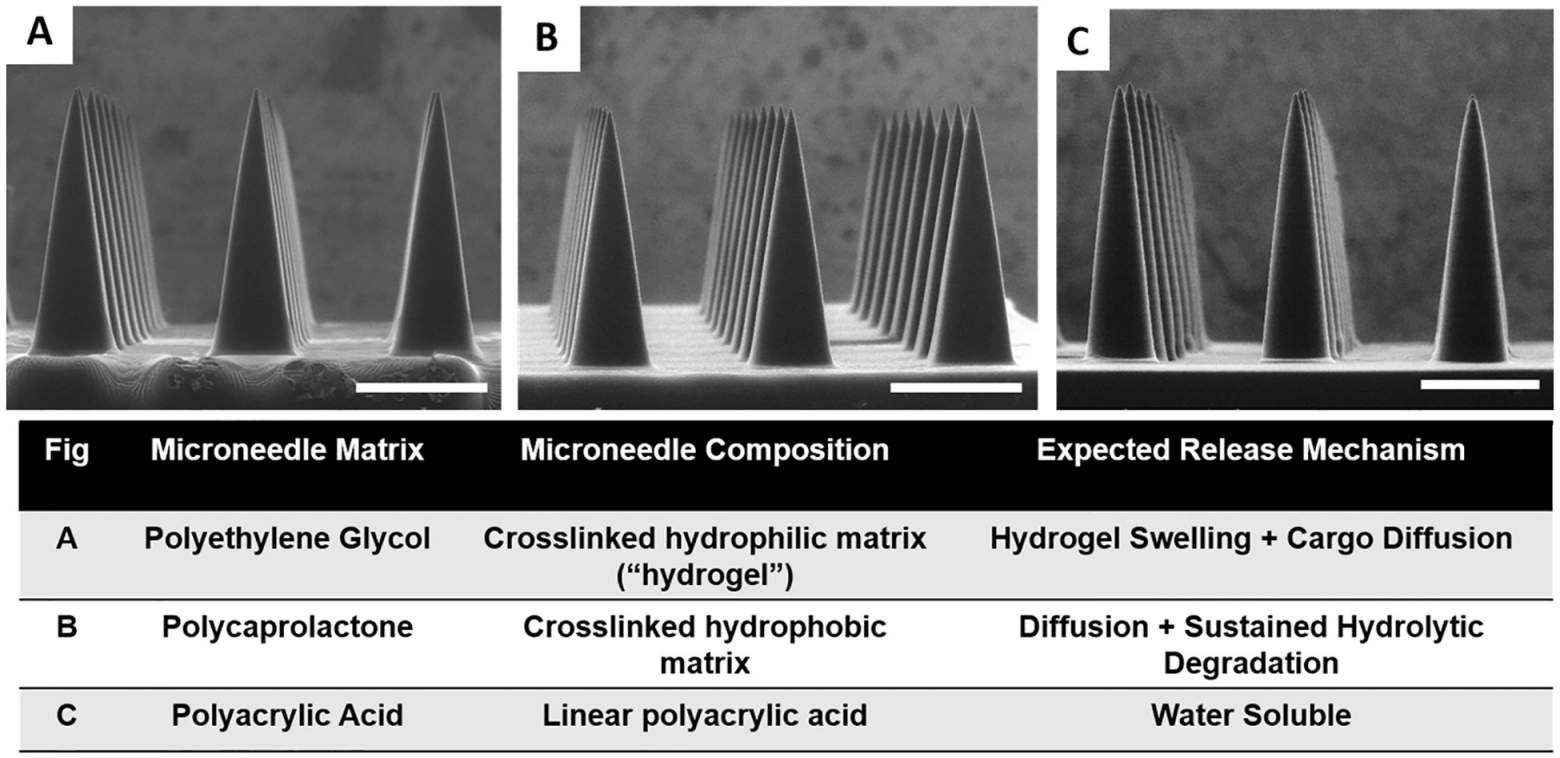
Biocompatible Microneedles. ESEM images of A) Polyethylene glycol B) Polycaprolactone and C) Polyacrylic acid microneedles measuring approximately 1000μm in height and 333μm in width. Starting resin, resulting microneedle composition, and expected cargo release mechanism for biocompatible microneedles are also provided. Scale bars measure 500μm.

Using this technique, fabrication of CLIP microneedles composed of PEG, PCL, and PAA was achieved (Fig 5) and the morphology of the CLIP microneedles was found to be very consistent between resins. These microneedles measure approximately 1000μm in height with an AR of 3, with all microneedles measuring within ±10% of their intended dimensions S2 Table. Tip radii for these biocompatible microneedles measure less than approximately 10μm, with fabrication times under 10 minutes per patch.

To confirm that these microneedle compositions provide an opportunity to tailor therapeutic release rates, each microneedle composition was loaded with rhodamine B base as a fluorescent drug surrogate (S8 Fig). Release of rhodamine B base into phosphate buffered saline (PBS) was assessed over a period of seven days (S9 Fig). PCL and PEG microneedles released 0.5wt% and 5wt% of loaded rhodamine over one week, respectively, whereas PAA microneedles released all of the loaded rhodamine within 30 minutes in solution. In order to confirm that PAA microneedles are completely dissolvable, rhodamine containing PAA microneedles on a PCL backing were imaged before and after submersion in aqueous media. Complete dissolution of the rhodamine containing PAA microneedles is observed within 15 minutes, leaving behind the water-insoluble PCL backing (S10 Fig). These results indicate that CLIP microneedle matrices can be easily altered to tune cargo release rates.

### Skin Penetration and Release of Fluorescent Drug Surrogate

**Fig 6:**
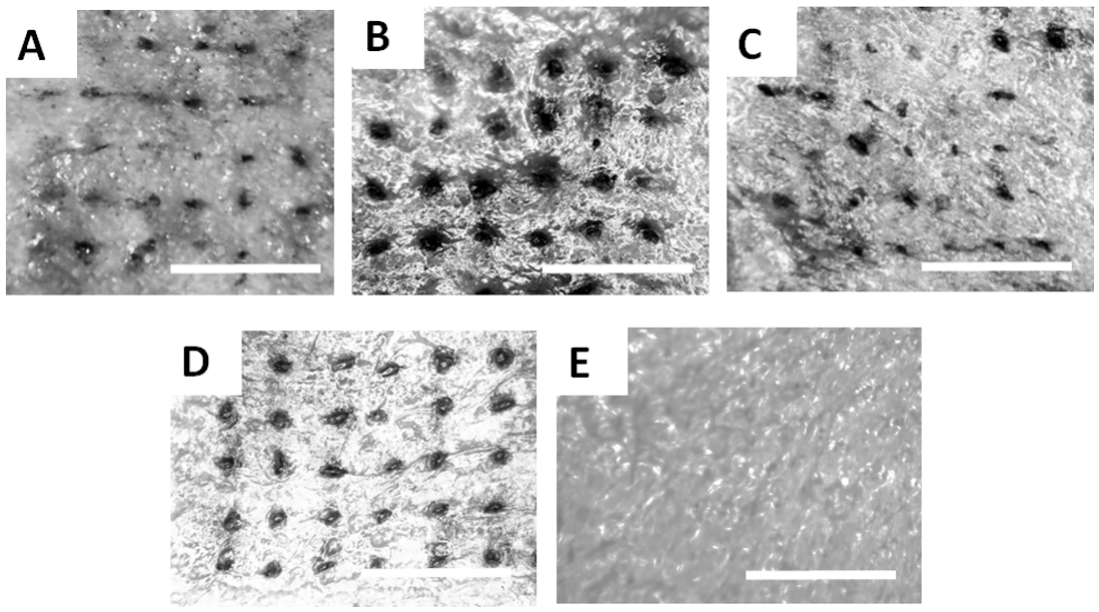
Skin Insertion Tests. Sites of skin penetration from CLIP Microneedle arrays made of A) PCL B) TMPTA C) PEG and D) Polyacrylic acid on murine skin can be visualized using a tissue marking dye. E) No insertion sites are visualized on a piece of control skin to which no microneedles were applied. Scale bars measure 1mm.

The ability of CLIP microneedles to puncture the skin and deliver a therapeutic cargo was assessed using ex-vivo murine skin. Murine skin was selected for model continuity with upcoming *in vivo* studies. Microneedles of four different compositions (TMPTA, PAA, PCL, and PEG) were applied to murine skin *ex vivo* by pressing firmly on the back of the microneedle patches with the thumb for 10 seconds. The microneedle patches were then removed and a green tissue marking dye that selectively marks sites of skin penetration was applied. All four microneedle compositions were observed to successfully breach murine skin (Fig 6A–6D), whereas no sites of penetration were observed on untreated skin (Fig 6E). Some qualitative differences in the penetration efficacy of the four different microneedle compositions could be observed. For example, the TMPTA and PAA microneedles appear to produce larger sites of penetration within the skin than the PCL and PEG microneedles, perhaps suggesting deeper penetration into the skin due to the superior mechanical properties. Nevertheless, these results indicates that all four microneedle compositions exhibit sufficient strength to pierce murine skin.

**Fig 7:**
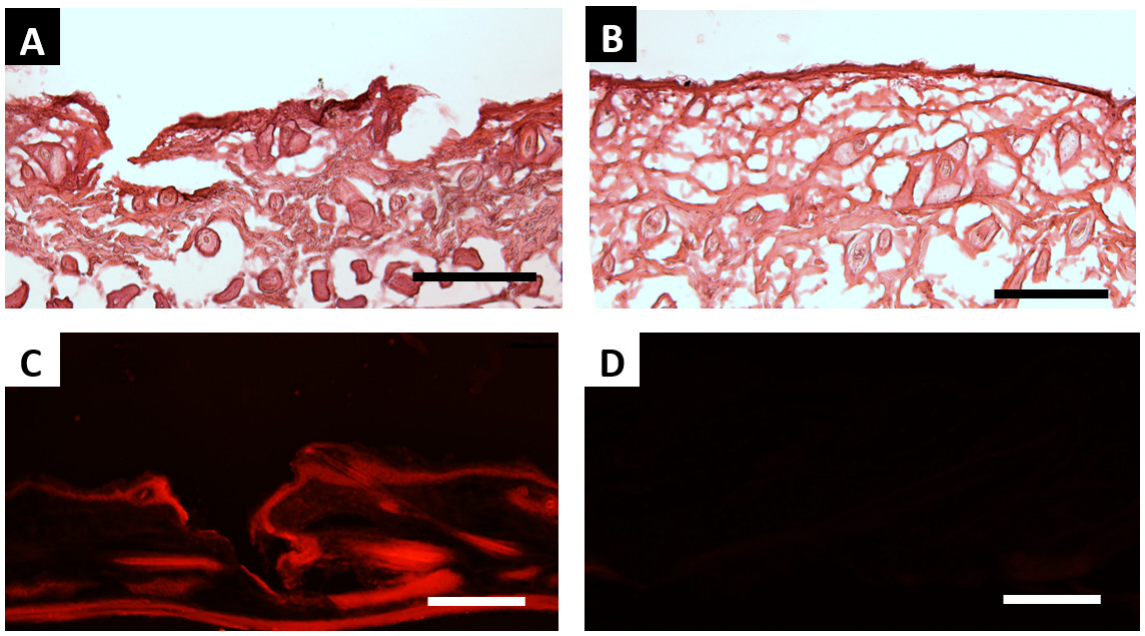
Ex-Vivo Skin Penetration and Dye Release. H&E stained skin sections show A) epidermal breach upon application of PAA microneedles but B) no epidermal breach in untreated control. C) The application of rhodamine containing polyacrylic acid microneedles releases rhodamine into the skin. D) No fluorescence is visualized in sections to which no microneedles were applied. All scale bars measure 100μm.

As further confirmation that CLIP microneedles puncture the stratum corneum, PAA microneedles were applied to additional samples of murine skin *ex-vivo*. These skin samples were then fixed, cryosectioned and stained with hematoxalin and eosin. Microneedle-induced disruption of the stratum corneum can be observed in Fig 7A, whereas untreated skin remained intact (Fig 7B).

To assess drug release, rhodamine was incorporated into polyacrylic acid microneedles as a fluorescent drug surrogate. Microneedles were applied to murine skin *ex-vivo* and allowed to dissolve within the skin for a period of 30 minutes. Skin samples were then fixed, cryosectioned, and visualized with a fluorescence microscope. Rhodamine could be observed within the treated skin (Fig 7C), whereas no fluorescence could be observed in untreated sections (Fig 7D). Together, these results suggest that CLIP microneedles can effectively deliver a drug surrogate into the skin.

Even though PAA CLIP microneedles dissolve to release a fluorescent drug surrogate into murine skin within 30 minutes, it was clear that the entirety of the microneedle did not dissolve in skin (S1 Fig). Therefore, we aimed to improve the efficiency of cargo delivery to the skin by localizing cargo to a water soluble tip. Janus microneedles composed of two distinct materials- a water insoluble PCL base containing rhodamine and a water soluble PAA tip containing fluorescein were fabricated to demonstrate that the fluorescein fluorescent drug surrogate could be localized to the microneedle tip. Fabrication of Janus microneedles was achieved by exchanging the resin in the middle of the production process. The microneedle base was first fabricated using the PCL resin before pausing the build. The support plate was then lifted above the residual resin pool was removed and replaced with acrylic acid resin. The remainder of the microneedle tip was then fabricated using the AA resin.

**Fig 8:**
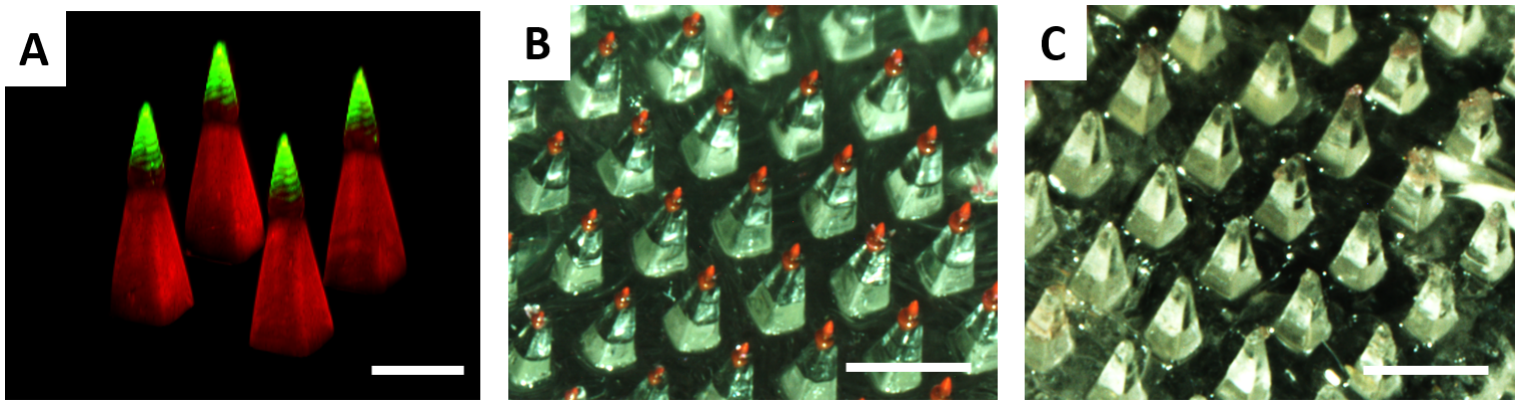
Tip loaded microneedles enable complete cargo delivery. A) Confocal micrograph of a Janus microneedle, where the microneedle base is composed of polycaprolactone encapsulating rhodamine and the microneedle tip is composed of polyacrylic acid encapsulating fluorescein. The rhodamine channel is given in red and the fluorescein channel is given in green; the overlay is displayed in yellow. Janus microneedle with a water soluble rhodamine containing tip B) before and C) after application to murine skin. Scale bars measure 500μm (A) and 1mm (B-C)

A confocal micrograph of the fabricated Janus microneedles is given in Fig 8A. The rhodamine channel is displayed in red and the fluorescein channel is displayed in green. The lack of overlap between the two fluorescent channels indicates that fluorescein was successfully localized to the microneedle tip.

In order to determine whether localizing cargo to the tip improves cargo delivery, Janus microneedles composed of a rhodamine containing PAA tip and a blank PCL base were fabricated and applied to murine skin *ex vivo* using firm thumb pressure for a period of 30 minutes. Visualization of the microneedle patch before and after application to murine skin (Fig 8B and 8C) demonstrates that the entirety of the rhodamine containing tip dissolved in the skin, resulting in complete release of cargo. Therefore, the CLIP process enables cargo to be easily localized to the microneedle tip to maximize the efficiency of delivery to the skin.

Together, these results indicate that CLIP can be used to produce microneedles from derivatives of widely used biocompatible polymers capable of controlling the release of cargo into the body.

## Discussion

The current focus of our research is harnessing the CLIP process to rapidly prototype microneedle arrays for direct application to the skin. CLIP provides unmatched flexibility over microneedle design; a nearly unlimited design space available with little to no process optimization required. Further, although it is somewhat difficult to compare the speed of CLIP to existing methods, we estimate that CLIP is up to 400-1600X faster than silicon etching techniques used to fabricate microneedle masters, which are typically accomplished at 1–5 μm/min.[9,41] Rapid prototyping enables a number of different parameters, such as size, shape, and composition, to be rapidly and systematically investigated in a high throughput manner. We anticipate that the benefits afforded by the CLIP technology will have an immediate impact by accelerating preclinical research. For example, that the role of microneedle shape and material properties on skin penetration could be clearly elucidated using this technique. Targeting specific cell populations within the skin, such as basal cell carcinomas, would also be possible by carefully controlling microneedle length and depths of penetration. A clear understanding of how microneedle design and formulation parameters affect drug pharmacokinetic profiles could also be easily garnered using CLIP microneedle technology.

The application of CLIP microneedle arrays to therapeutic applications requires further investigation. In particular, methods for encapsulating and stabilizing therapeutic compounds and the biocompatibility of resulting microneedle devices still need further development. Careful assessment of the safety profile of CLIP microneedle devices is necessary. Residual unreacted acrylic monomers and oligomers have been associated with toxicity;[42] therefore, ensuring high extents of reaction completion during the photopolymerization process and/or complete removal of residual monomer is critical to future device development. The nature of the degradation products should also be further investigated. For example, we expect that polymerization of the PCL-tMa would produce a final product that is a combination of degradable monomeric units and nondegradable crosslinks. It will be important to characterize the molecular weight of degradation products to ensure successful elimination from the body.

The true strength of CLIP microneedle technology, demonstrated herein, is the ability to rapidly alter microneedle design. While we anticipate that successful formulation will enable CLIP microneedles to be directly utilized for therapeutic applications, there is also a powerful opportunity to combine CLIP technology with existing micromolding techniques. The adaptability and speed of CLIP microneedle fabrication make it an unparalleled technique for generating microneedle master templates, which could be replicated using polydimethylsiloxane (PDMS) molds. This PDMS mold could then be filled using proven micromolding techniques, which effectively stabilize a wide variety of therapeutics for delivery[1–2,11] (but with substantially longer fabrication times than the CLIP technique presented here).

## Conclusions

Together, these results demonstrate the ability to use CLIP to rapidly generate microneedles of various sizes, shapes, aspect ratios, spacings, and compositions. CLIP microneedles punctured murine skin *ex vivo* and released the fluorescent drug surrogate rhodamine. The work presented here is the fastest and most versatile microneedle prototyping scheme to date, to our knowledge, and provides a tunable platform for the systematic study of numerous parameters associated with transdermal delivery via microneedles. These parameters include, but are not limited to, microneedle geometry, material properties (such as strength and elasticity), therapeutic incorporation and release and their resulting effect on therapeutic efficacy.

## Supporting Information

S1 Fig Overview of the additive manufacturing process. A computer model is computationally sliced into individual layers. Each two dimensional layer is stacked on top of the previous layer to create the desired three-dimensional part
(TIF)

S2 Fig Difference between stereolithography and CLIP. (TIF)

S3 Fig CLIP eliminates the trade-off between slice thickness and fabrication time by eliminating separation and realignment steps, enabling rapid production of high resolution structures (TIF)

S4 Fig TMPTA microneedles produced with varying light intensity. Microneedles were produced using A) 2mW/cm^2^, B) 5mW/cm^2^, C) 8mW/cm^2^, D) 11mW/cm^2^, and E) 14mW/cm^2^ of UV light. Build speed was held constant at 100mm/hr. Scale bars measure 500μm.
(TIF)

S5 Fig Dimensions of TMPTA microneedles produced with varying light intensity. Microneedles were produced using 1.4mW/cm^2^, 3.4mW/cm^2^, 5.4mW/cm^2^, 7.4mW/cm^2^ and 9.5mW/cm^2^ of UV light in triplicate and measured (total n = 9, n = 3 individual microneedles from each array). The height and width of the input CAD file are marked with dashed lines.
(TIF)

S6 Fig Reaction scheme and ^1^H NMR Spectrum for PCL-trimethacrylate synthesis. A) PCL was functionalized by reacting hydroxyl groups from a PCL-triol with methacryloyl chloride B) ^1^H NMR spectrum confirms methacrylate functionalization with peaks at 6.08 (c), 5.54 (b) and 1.93 ppm (d). Degree of functionalization was determined to be 89% by comparing the peak areas corresponding to the vinyl protons (c and b, 6.08 and 5.54 ppm) to the protons of the methyl group in the PCL backbone (a, 0.89 ppm).
(TIF)

S7 Fig Stereolithographic working curve and resulting parameters. **A)** The cure depth of microneedle resins as a function of applied dosage B) Absorption coefficient and critical exposure of microneedle resins determined from the working curves in A
(TIF)

S8 Fig Rhodamine loaded CLIP MNs. Incorporation of rhodamine does not alter structure of A) PEG, B) PCL or C) PAA MNs characterized by ESEM. Rhodamine distributes throughout D) PEG, E) PCL, and F) PAA MNs needles visualized via confocal microscopy. The rhodamine channel is displayed in purple. Scale bars measure 500μm.
(TIF)

S9 Fig Rhodamine release rates in phosphate buffered saline. Rates of rhodamine release from A) PEG, PCL and B) PAA MNs loaded with 0.1wt% rhodamine
(TIF)

S10 Fig Dissolution of rhodamine containing PAA microneedles. PAA microneedles completely dissolve within 15 minutes in PBS. Scale bars measure 1 mm.
(TIF)

S11 Fig PAA microneedle before and after application to murine skin. ESEM of microneedle A) before and B) one hour after application to murine skin. Partial dissolution of the needle is observed, suggesting incomplete insertion into the skin. Scale bars measure 1mm.
(TIF)

S1 File Additional Methods. Methods for working curve determination and rhodamine release studies.
(DOCX)

S2 File Raw Data. Raw data for all microneedle dimensions, working curves, and release studies.
(XLSX)

S1 Table Dimensions of TMPTA microneedles of varying heights after identification of appropriate build conditions. (TIF)

S2 Table Biodegradable microneedle dimensions and print times Dimensions, print times, and tip radii of biodegradable microneedles (n = 9) shown in Fig 5. All data are represented as mean ± standard deviation
(TIF)
