# Supplementary figures and images for: Single-Step Fabrication of Computationally Designed Microneedles by Continuous Liquid Interface Production
Source: PLoS One. 2016 Sep 8;11(9):e0162518. doi: 10.1371/journal.pone.0162518 (PMC5015976; doi:10.1371/journal.pone.0162518)

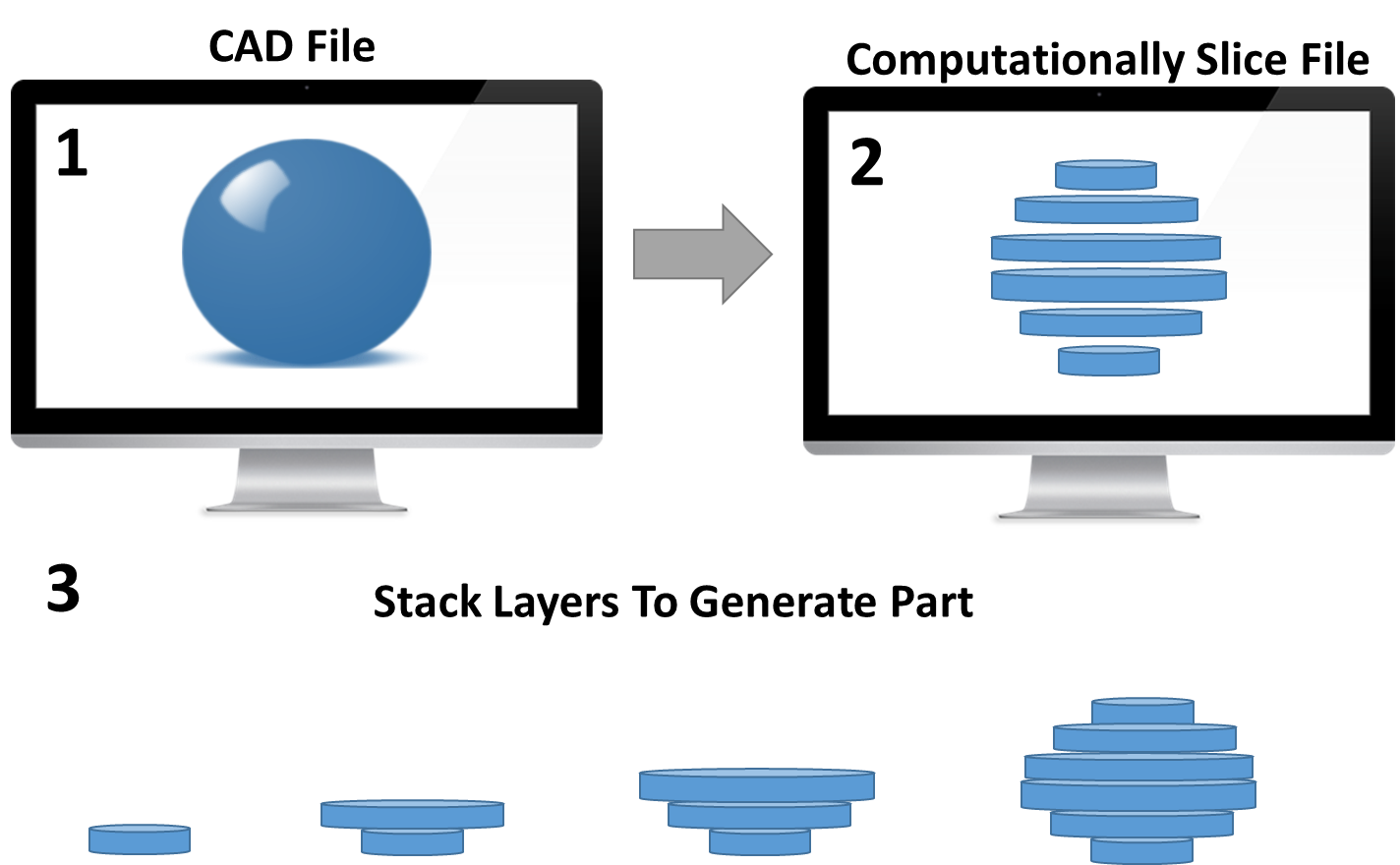

Supplement: S1 Fig — A computer model is computationally sliced into individual layers. Each two dimensional layer is stacked on top of the previous layer to create the desired three-dimensional part (TIF) [file pone.0162518.s001.tif]

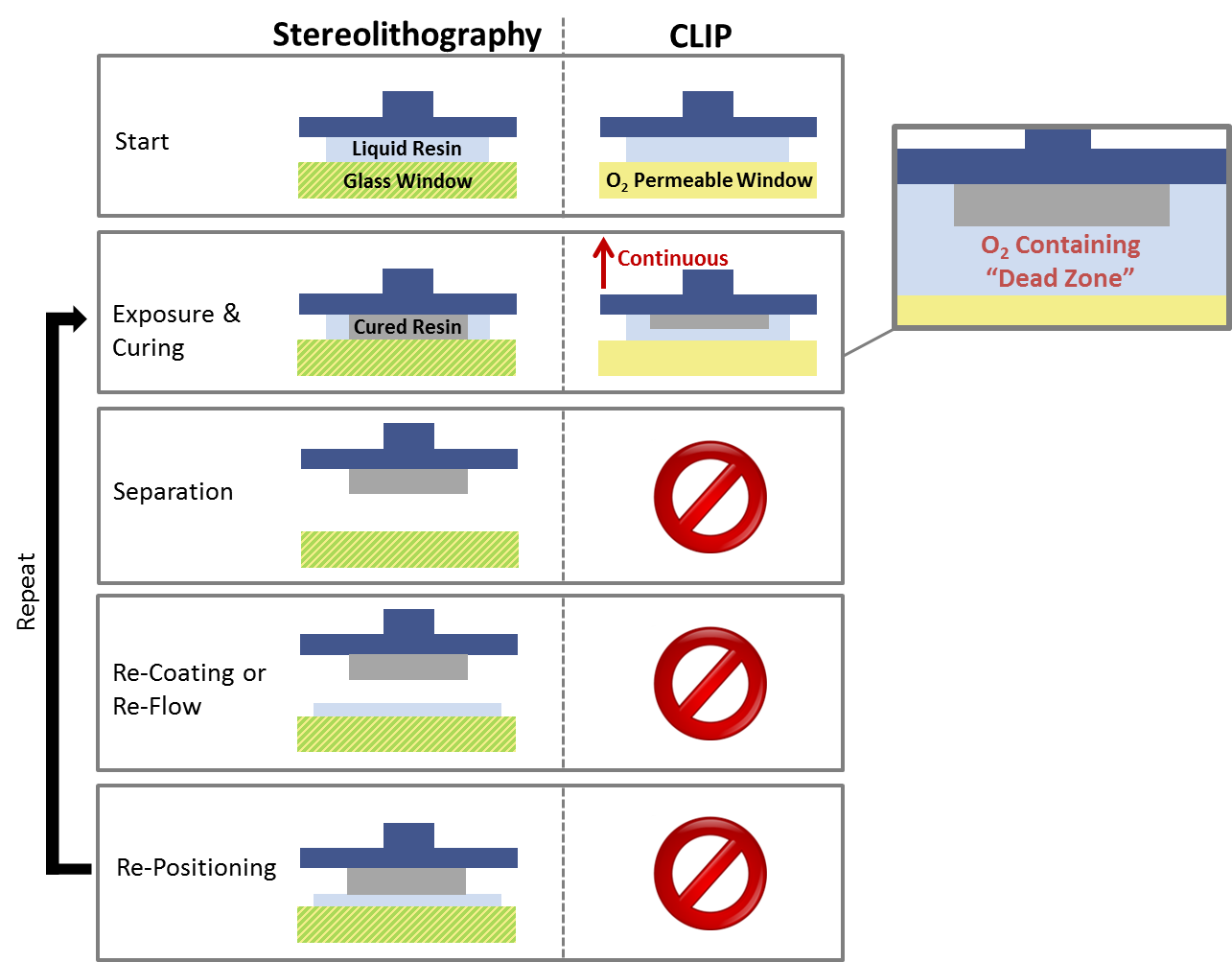

Supplement: S2 Fig — (TIF) [file pone.0162518.s002.tif]

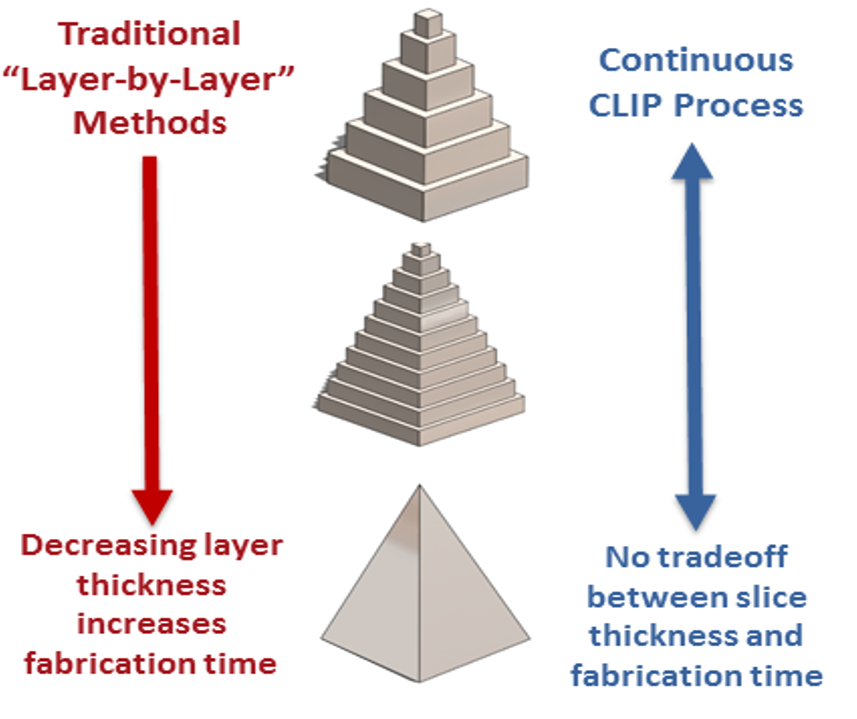

Supplement: S3 Fig — (TIF) [file pone.0162518.s003.tif]

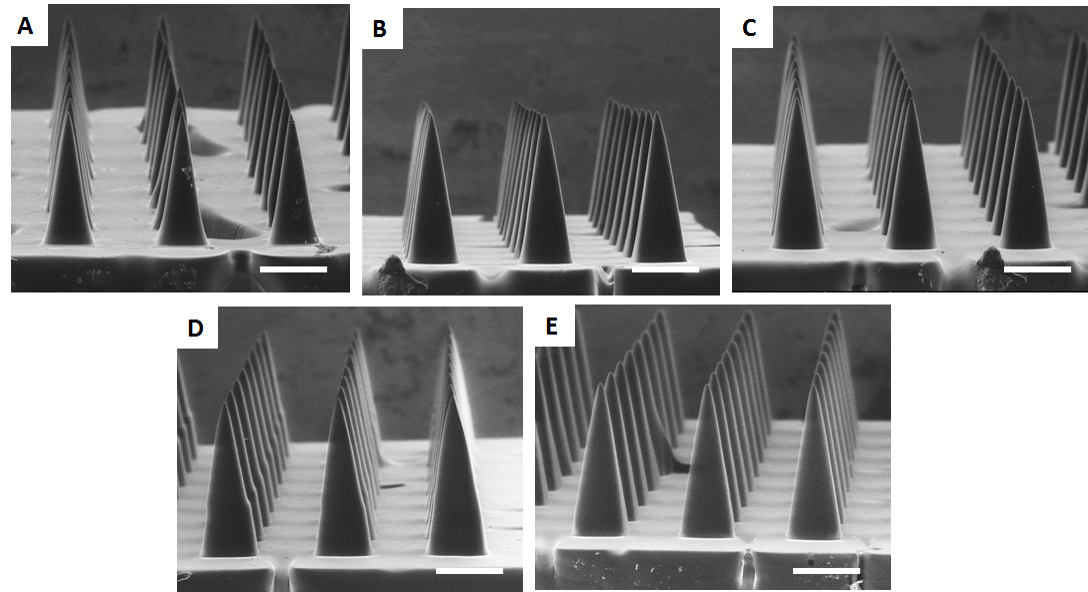

Supplement: S4 Fig — Microneedles were produced using A) 2mW/cm2, B) 5mW/cm2, C) 8mW/cm2, D) 11mW/cm2, and E) 14mW/cm2 of UV light. Build speed was held constant at 100mm/hr. Scale bars measure 500μm. (TIF) [file pone.0162518.s004.tif]

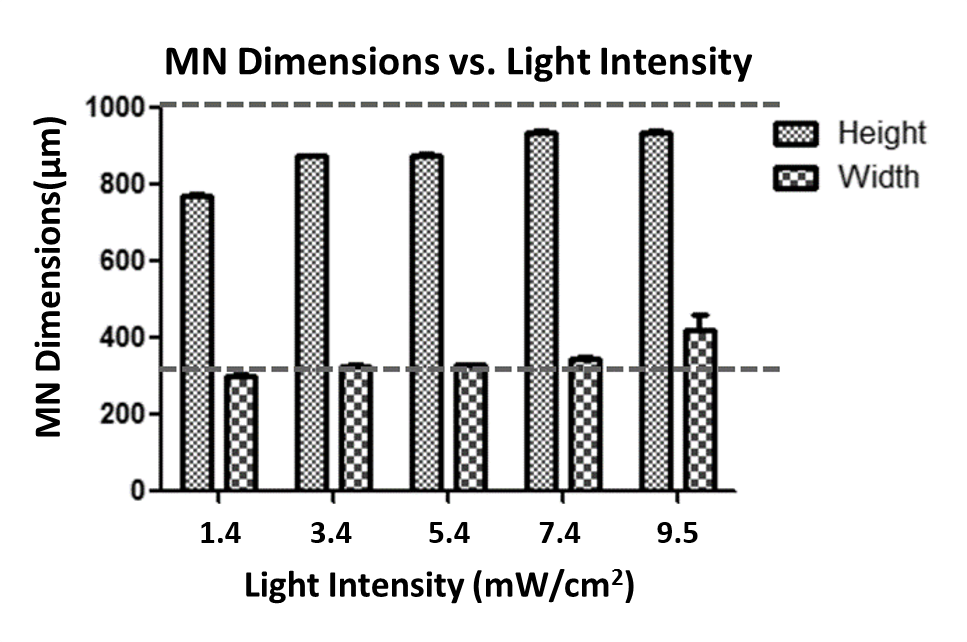

Supplement: S5 Fig — Microneedles were produced using 1.4mW/cm2, 3.4mW/cm2, 5.4mW/cm2, 7.4mW/cm2 and 9.5mW/cm2 of UV light in triplicate and measured (total n = 9, n = 3 individual microneedles from each array). The height and width of the input CAD file are marked with dashed lines. (TIF) [file pone.0162518.s005.tif]

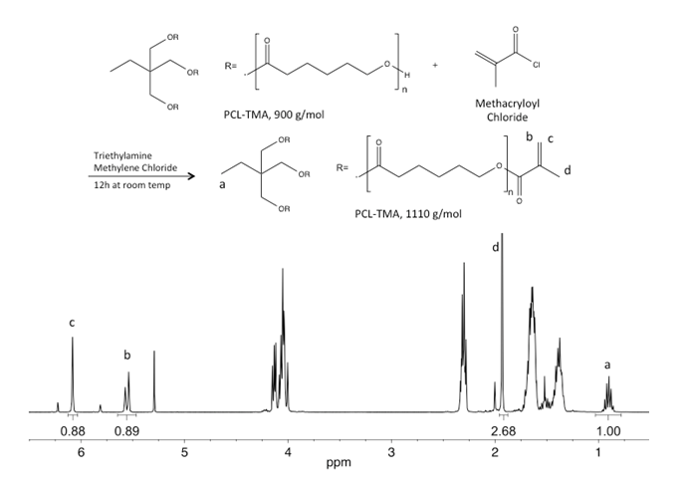

Supplement: S6 Fig — A) PCL was functionalized by reacting hydroxyl groups from a PCL-triol with methacryloyl chloride B) 1H NMR spectrum confirms methacrylate functionalization with peaks at 6.08 (c), 5.54 (b) and 1.93 ppm (d). Degree of functionalization was determined to be 89% by comparing the peak areas corresponding to the vinyl protons (c and b, 6.08 and 5.54 ppm) to the protons of the methyl group in the PCL backbone (a, 0.89 ppm). (TIF) [file pone.0162518.s006.tif]

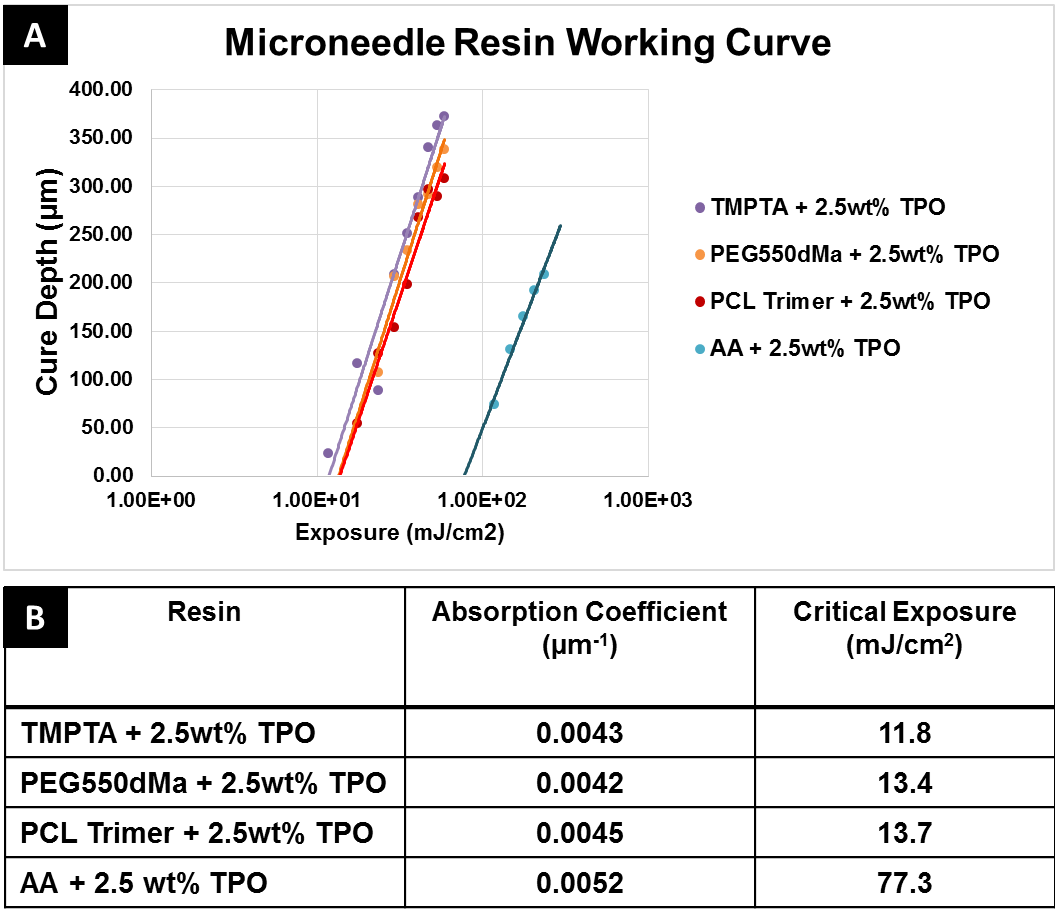

Supplement: S7 Fig — A) The cure depth of microneedle resins as a function of applied dosage B) Absorption coefficient and critical exposure of microneedle resins determined from the working curves in A (TIF) [file pone.0162518.s007.tif]

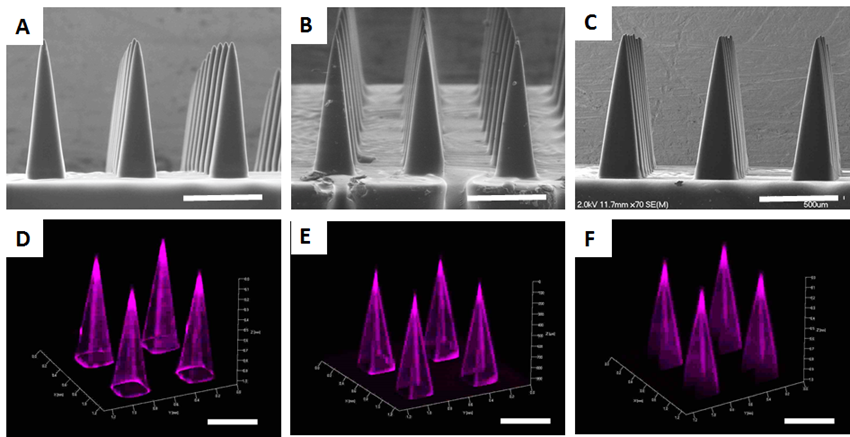

Supplement: S8 Fig — Incorporation of rhodamine does not alter structure of A) PEG, B) PCL or C) PAA MNs characterized by ESEM. Rhodamine distributes throughout D) PEG, E) PCL, and F) PAA MNs needles visualized via confocal microscopy. The rhodamine channel is displayed in purple. Scale bars measure 500μm. (TIF) [file pone.0162518.s008.tif]

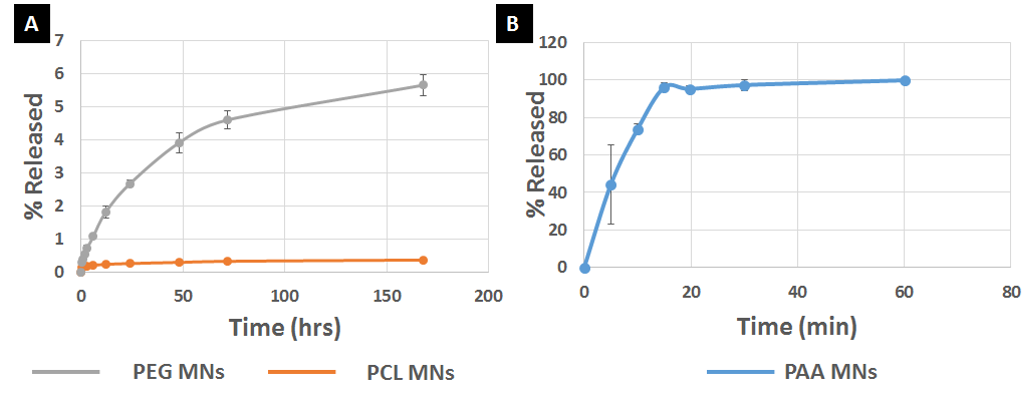

Supplement: S9 Fig — Rates of rhodamine release from A) PEG, PCL and B) PAA MNs loaded with 0.1wt% rhodamine (TIF) [file pone.0162518.s009.tif]

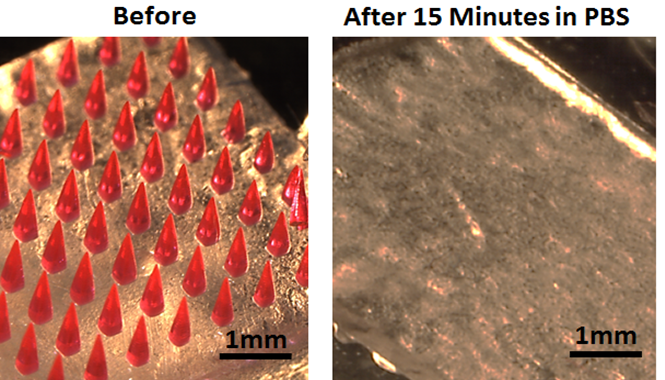

Supplement: S10 Fig — PAA microneedles completely dissolve within 15 minutes in PBS. Scale bars measure 1 mm. (TIF) [file pone.0162518.s010.tif]

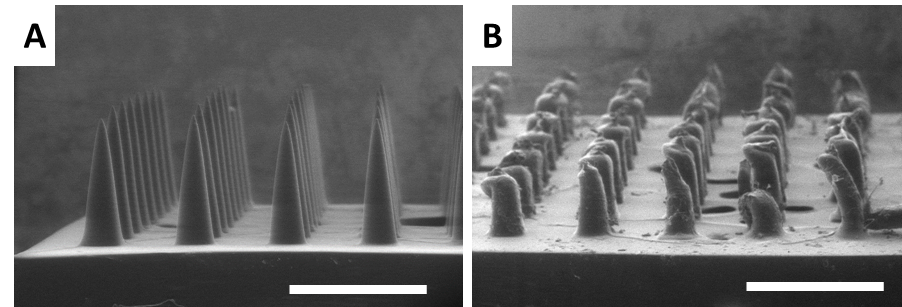

Supplement: S11 Fig — ESEM of microneedle A) before and B) one hour after application to murine skin. Partial dissolution of the needle is observed, suggesting incomplete insertion into the skin. Scale bars measure 1mm. (TIF) [file pone.0162518.s011.tif]

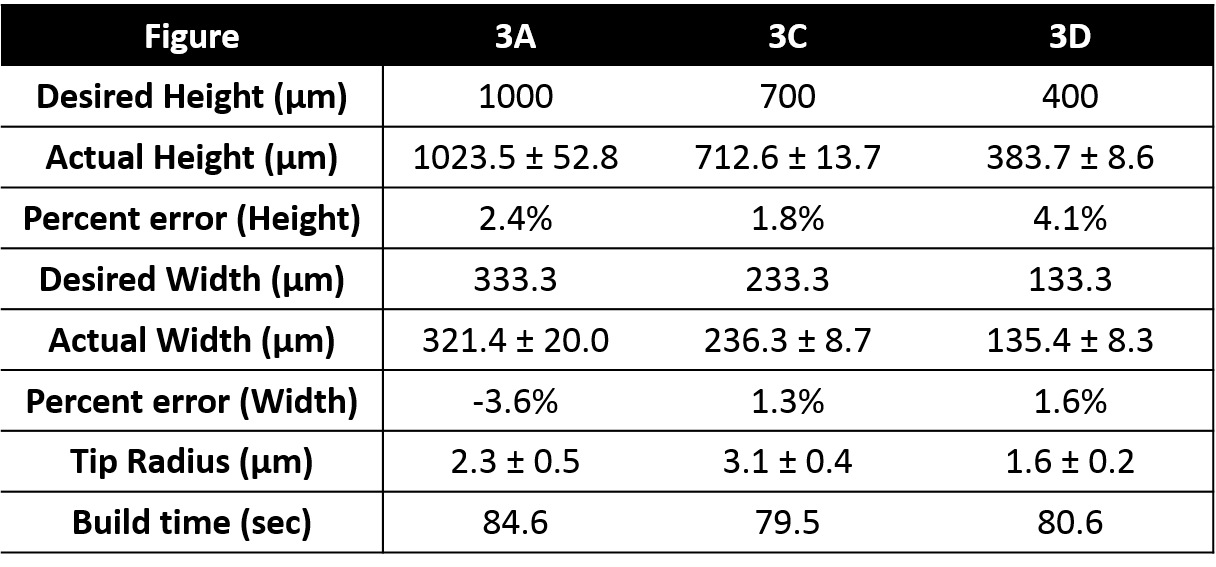

Supplement: S1 Table — (TIF) [file pone.0162518.s014.tif]

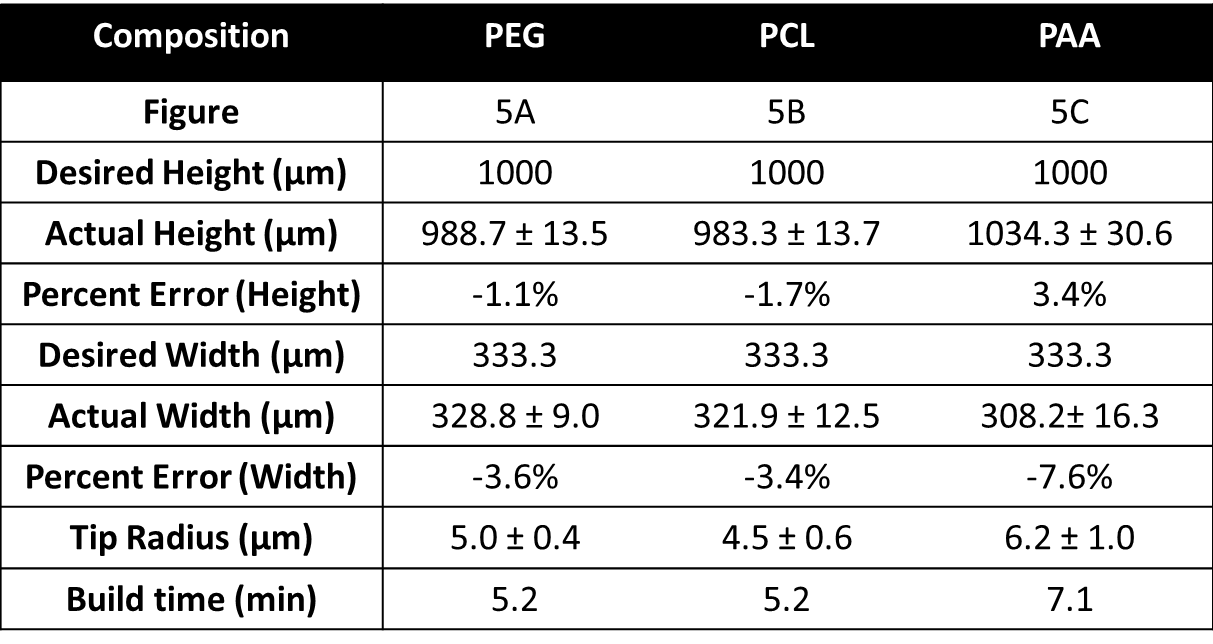

Supplement: S2 Table — All data are represented as mean ± standard deviation (TIF) [file pone.0162518.s015.tif]
