# Supplementary material for: Single-Step Fabrication of Computationally Designed Microneedles by Continuous Liquid Interface Production
Source: PLoS One. 2016 Sep 8;11(9):e0162518. doi: 10.1371/journal.pone.0162518 (PMC5015976; doi:10.1371/journal.pone.0162518)
Supplement: S1 File — Methods for working curve determination and rhodamine release studies. (DOCX) [file pone.0162518.s012.docx]

# Additional Methods

**Determination of Microneedle Build Parameters**

A stereolithographic working curve was utilized to determine the appropriate light intensity and draw speed required to fabricate CLIP Microneedles. The working curve defines the thickness of an individual frame resulting from exposure to a specific amount of light.

Briefly, a dot of resin was placed on a cover slip on top of the printer window and exposed to a specified amount of light, where exposure is defined as the product of light intensity (in mW/cm^2^) and exposure time (in seconds). The height of the resulting object was determined and plotted on a log-linear scale (Figure S1), where the best fit line between individual data points correlates with the equation

C_T_ $=\frac{1}{\alpha}ln(\frac{E}{E_{c}})$ (1)

where C_T_ is the thickness of a cured dot, α is the absorption coefficient of the resin in units of µm^-1^, E is exposure of an individual frame (the product of light intensity and exposure time, given in mJ/cm^2^), and $E_{c}$ is the critical exposure required to induce polymerization of the resin in units of mJ/cm^2^. The derivation of Equation (1) from the Beer-Lambert law is given in a number of stereolithography textbooks^37^ and applied to CLIP in Tumbleston et. al.^32^ The best fit line was determined via logarithmic fitting in Excel 2013. The absorption coefficient α and critical exposure $E_{c}$of the resins (as reported in Figure S3B) could then be determined from the slope and x intercept of the best fit line, respectively.

After empirically determining the exposure per frame appropriate for fabricating TMPTA microneedles, the exposure per frame necessary for fabricating microneedles in other resins could be identified by selecting the exposure that produces a cure thickness equivalent to TMPTA according to Equation (2) and (3), below.

$C_{T, TMPTA}=C_{T,ResinX}$ (2)

$1/\alpha_{TMPTA} ln\left( E_{TMPTA}/ E_{c, TMPTA} \right)=1/\alpha_{ResinX} ln(E_{Resin X} / E_{c, ResinX})$ (3)

During a continuous print, the applied exposure per frame (assuming constant slice thickness) is proportional to the applied light intensity divided by the build speed, given in mm/hr in the z direction, as shown in Equation (4)

$E_{applied}\propto\frac{Light Intensity}{Build Speed}$ (4)

Because the absorption coefficients of microneedle resins are approximately equivalent (see Figure S3), substituting into Equation (3) yields

$\frac{{Intensity}_{TMPTA}}{E_{c, TMPTA}\cdot{Speed}_{TMPTA}}=\frac{{Intensity}_{Resin X}}{E_{c, ResinX}\cdot{Speed}_{ResinX}}$ (5)

A build speed of 25 mm/hr was selected for fabrication of biodegradable microneedles to minimize the potential for defects due to resin flow into the build area. Therefore, the light intensity required for microneedle fabrication from an arbitrary Resin X could be calculated using Equation (6), below

${Intensity}_{ResinX}=\frac{{Intensity}_{TMPTA}\cdot E_{c,ResinX}\cdot{Speed}_{ResinX}}{{Speed}_{TMPTA}\cdot E_{c,TMPTA}}$ (6)

**Loading and Release of Rhodamine as a Fluorescent Drug Surrogate**

To model drug release profiles, all microneedle compositions were loaded with 0.1wt% Rhodamine B Base (Acros Organics). All microneedles were fabricated on a blank PEG base containing no rhodamine, fabricated with 1.2 mW/cm^2^ of light at 100 mm/hr. The resin was then postcured under a mercury lamp for 10 minutes. Resin was removed and replaced with resin containing rhodamine prior to fabricating the microneedles using the build parameters given in materials and methods. Confocal z stacks One microneedle patch was added to 1mL of phosphate buffered saline (PBS) in an Eppendorf tube (n=3 for each composition). The Eppendorf tubes were placed in a 37°C water bath. 0.5 mL of the supernatant was removed at each time point and replaced with an additional 0.5mL of PBS. Quantification of released rhodamine was performed by measuring fluorescence of the supernatant using a plate reader with excitation and emission of 544 and 590nm, respectively. Fluorescence was correlated to a standard curve to determine rhodamine mass released, then taken as a percent of theoretical rhodamine loading, calculated from microneedle volume and density.
